# Palladium Promoted Production of Higher Amines from a Lower Amine Feedstock

**DOI:** 10.1007/s10562-016-1902-7

**Published:** 2017-02-14

**Authors:** Yufen Hao, Fernando Cárdenas-Lizana, Mark A. Keane

**Affiliations:** Chemical Engineering, School of Engineering and Physical Sciences, Heriot-Watt University, Edinburgh, EH14 4AS Scotland, UK

**Keywords:** Secondary amine, Tertiary amine, Pd/Al_2_O_3_, Pd/C, Catalyst beds in series

## Abstract

**Abstract:**

The catalytic (Pd/Al_2_O_3_ and Pd/C; mean Pd size 2.5–3.0 nm from (S)TEM analysis) synthesis of di-butylamine (DBA) and tri-butylamine (TBA) from mono-butylamine (MBA) and DBA, respectively, in continuous gas phase operation is demonstrated. Exclusive production of DBA (from MBA) has been established over both catalysts where 453 ≤ *T* ≤ 523 K (∆E_a_ = 79 kJ mol^−1^). Greater activity for Pd/C is associated with higher levels of surface acidity (from NH_3_ chemisorption/TPD) and spillover hydrogen (from H_2_ TPD). Reaction of DBA over both catalysts when configured in series delivered full selectivity to TBA. Our results establish a novel clean alternative route for the continuous production of higher (secondary and tertiary) amines.

**Graphical Abstract:**

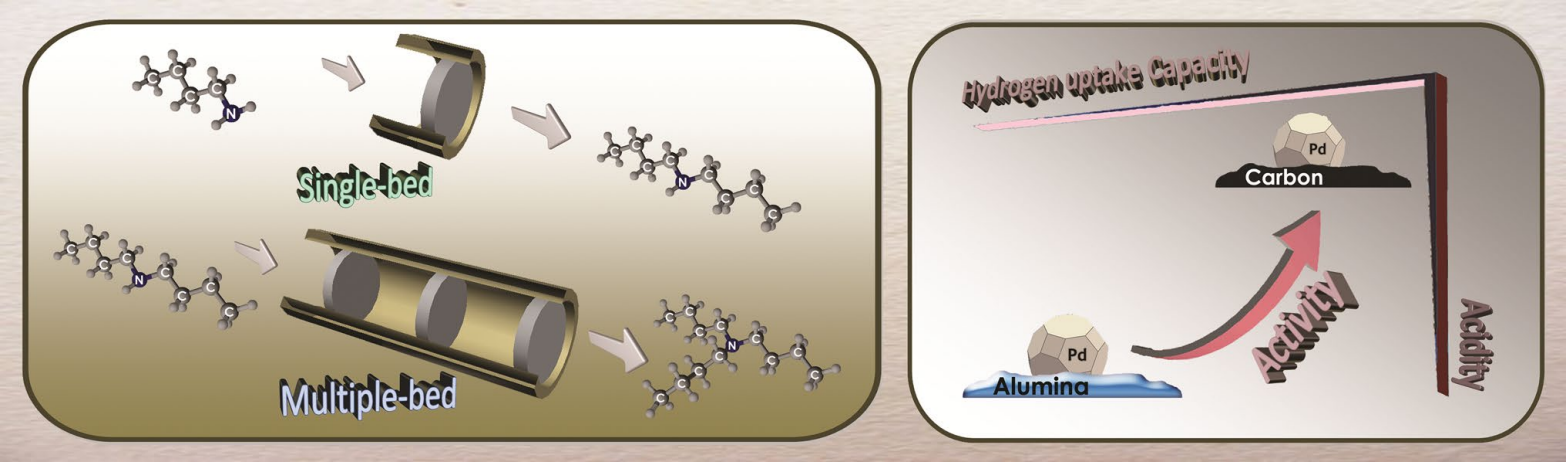

## Introduction

**Fig. 1:**
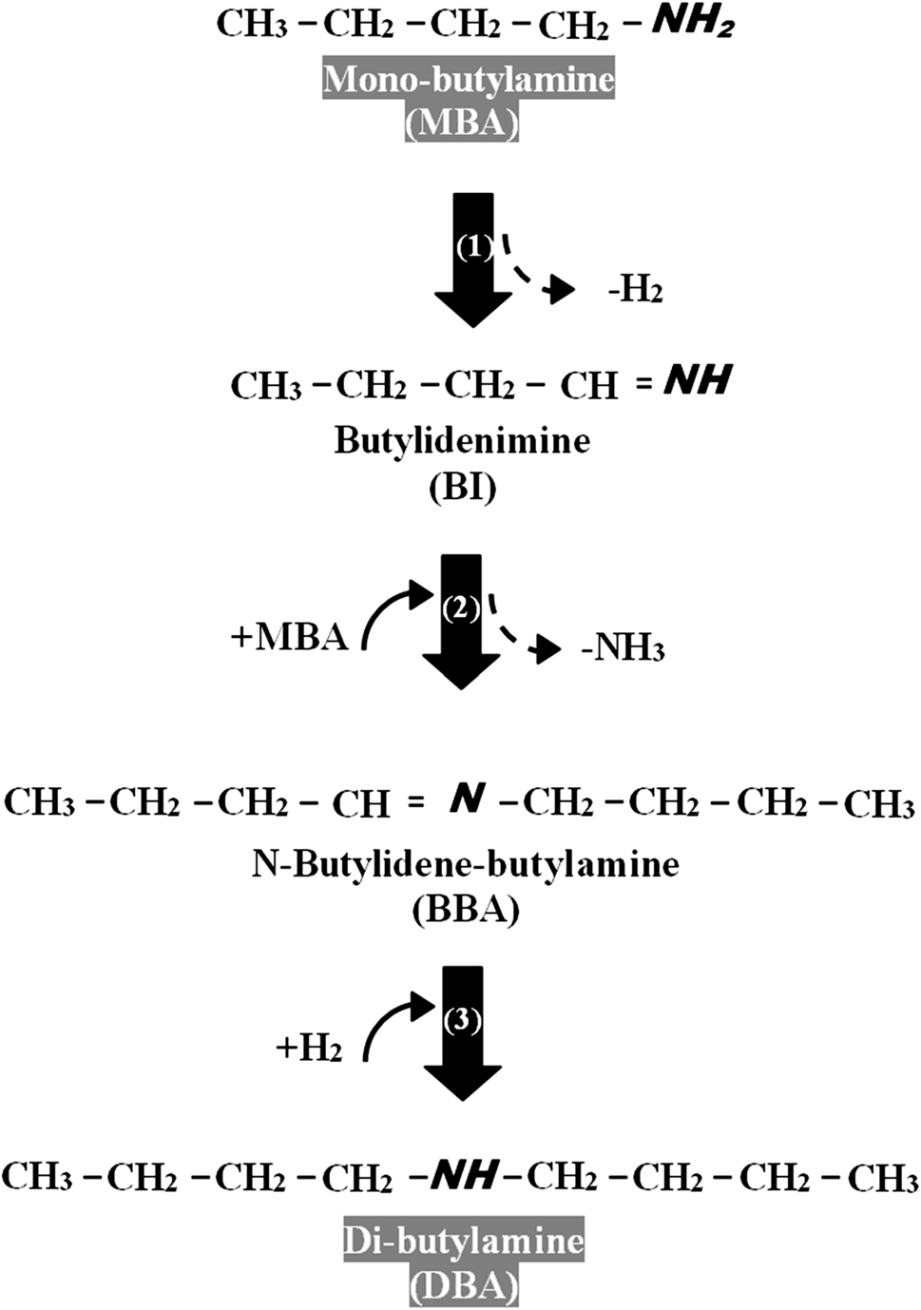
Schematic showing the reaction pathways associated with the conversion of mono-butylamine (MBA) to di-butylamine (DBA)

Higher (secondary and tertiary) amines are commercially important in the production of a range of chemical products [1, 2] used in drug production [3] and as solvents in extraction processes [2]. Standard synthesis involves (i) *N*-alkylation of primary amines with alkyl halides or alcohols [1, 4, 5], (ii) reduction of imines using reducing agents (e.g. NaCNBH_3_) in liquid batch mode [3] or hydrogenation of nitriles over supported Pt [6, 7] and Pd [8]. These methods are non-selective and produce amine mixtures. Moreover, they use toxic agents and require complex extraction of the target product with multiple separation/purification steps. Conversion of an amine feedstock to a higher amine product has been considered to a limited extent in the literature and the reaction mechanism is poorly understood. Taking mono-butylamine (MBA) as a model reactant (Fig. 1), dehydrogenation (step 1) generates a reactive butylidenimine (BI) intermediate, which readily reacts with MBA (step 2) to form *N*-butylidene-butylamine (BBA) with the elimination of 1 mol NH_3_. BBA can be hydrogenated (step 3) to di-butylamine (DBA). Tertiary amine synthesis from secondary amines has only been reported by homogeneous catalysis [RuCl_3_·xH_2_O and P(C_6_H_5_)] in one patent [9] and we could not find any published reaction mechanism for di-amine to tri-amine transformation. The limited reports on primary amine condensation have employed homogenous catalysts [3, 10–14] that are difficult to recover and reuse. An efficient amine condensation system utilising reusable heterogeneous catalysts in continuous mode at ambient pressure as proposed in this work represents a significant advancement in terms of cleaner processing.

Work to date on the condensation of primary amines over heterogeneous systems suffers from data irreproducibility in terms of temperature control associated with microwave irradiation (of MBA over Pt/C mixed with alumina powder [15]) and the formation of significant amounts of undesired imine by-product (over Cu/Al_2_O_3_ [16] and Pt/C [10]). Given the established performance of Pd catalysts in dehydrogenation [17–19] and hydrogenation [20–22], critical steps in higher amine synthesis (see Fig. 1), we have adopted Pd/C and Pd/Al_2_O_3_ as suitable catalyst candidates for this process. Activated carbon [23, 24] and alumina [25–27] supports bear acid sites that favour condensation to generate higher amines [28, 29], as has been shown for acid catalysts (e.g. K-10 montmorillonite [30, 31]). Moreover, electron transfer between the carrier and Pd phase can influence reactant activation and impact on catalytic performance [32] but this effect has not been considered in higher amine production. In this study, we evaluate the feasibility of secondary and tertiary amine production from lower amines in continuous gas phase operation. We compare the catalytic action of Pd/C and Pd/Al_2_O_3_ and propose a reaction mechanism based on our results. We demonstrate that the use of catalyst beds in series facilitates full selectivity to the higher amine at elevated rates.

## Experimental

### Catalyst Characterisation

Commercial (1% w/w) Pd/C and Pd/Al_2_O_3_ catalysts were obtained from Sigma-Aldrich. The Pd content was measured by inductively coupled plasma-optical emission spectrometry (ICP-OES, Vista-PRO, Varian Inc.) from the diluted extract in HF. Catalyst activation by temperature programmed reduction (TPR), H_2_ and NH_3_ chemisorption, temperature programmed desorption (TPD) and specific surface area (SSA) measurements were carried out in the CHEM-BET 3000 (Quantachrome) unit equipped with a thermal conductivity detector (TCD) for continuous monitoring of gas composition and the TPR Win™ software for data acquisition/manipulation. Samples (0.05–0.1 g) were loaded in a U-shaped Quartz cell (3.76 mm i.d.), outgassed for 30 min and the total SSA recorded in a 30% v/v N_2_/He flow with undiluted N_2_ (BOC, 99.9%) as internal standard. Two cycles of N_2_ adsorption–desorption were employed using the standard single point BET method. TPR was conducted in 17 cm^3^ min^−1^ (Brooks mass flow controlled) 5% v/v H_2_/N_2_ at 2 K min^−1^ to 573 K [32]. Samples were swept with 65 cm^3^ min^−1^ N_2_ for 1.5 h, cooled to ambient temperature and subjected to H_2_ (BOC, 99.99%) or NH_3_ (BOC, 99.98%) pulse (50–1000 µl) titration. The samples were thoroughly flushed in N_2_/He (65 cm^3^ min^−1^) to remove weakly bound H_2_ or NH_3_ and subjected to TPD at 10–50 K min^−1^ (in 65 cm^3^ min^−1^ N_2_) to 950–1200 K. SSA and H_2_/NH_3_ uptake/release values were reproducible to within ±5% and the values quoted represent the mean. Palladium particle morphology (size and shape) was determined by transmission (JEOL JEM 2011 TEM unit) and scanning transmission (JEOL 2200FS field emission gun-equipped TEM unit) electron microscopy, employing Gatan DigitalMicrograph 1.82 for data acquisition/manipulation. Samples for analysis were crushed and deposited (dry) on a holey carbon/Cu grid (300 Mesh). Up to 800 individual Pd particles were counted for each catalyst and the surface area-weighted metal diameter (*d*_(S)TEM_) calculated from

1 $$d_{\text{(S)TEM}}=\frac{\sum_{\text{i}}n_{\text{i}}d_{\text{i}}^{3}}{\sum_{\text{i}}n_{\text{i}}d_{\text{i}}^{2}}$$

where *n*_i_ is the number of particles of diameter *d*_i_. X-ray photoelectron spectroscopy (XPS) analyses were conducted on an Axis Ultra instrument (Kratos Analytical) under ultra-high vacuum conditions (<10^−8^ Torr) using a monochromatic Al Kα X-ray source (1486.6 eV). The source power was maintained at 150 W and the emitted photoelectrons were sampled from a 750 × 350 µm^2^ area at a take-off angle = 90°. The analyser pass energy was 80 eV for survey spectra (0–1000 eV) and 40 eV for high resolution spectra (Pd 3*d*_5/2_ and 3*d*_3/2_). The adventitious carbon 1*s* peak was calibrated at 284.5 eV and used as an internal standard to compensate for charging effects.

### Catalytic Procedure

Reactions (of MBA and DBA, Sigma-Aldrich, ≥99%) were conducted in situ, immediately after catalyst activation, under atmospheric pressure over the temperature range 453–523 K in a fixed bed vertical glass reactor (i.d. = 15 mm). The reactant was delivered at a fixed calibrated flow rate to the reactor via a glass/Teflon air-tight syringe and Teflon line using a microprocessor controlled infusion pump (Model 100 kd Scientific). A layer of borosilicate glass beads served as preheating zone, ensuring the reactants were vaporised and reached reaction temperature before contacting the catalyst bed. Isothermal conditions (±1 K) were maintained by diluting the catalyst bed with ground glass (75 µm); the ground glass was mixed thoroughly with catalyst before insertion in the reactor. Reaction temperature was continuously monitored using a thermocouple inserted in a thermowell within the catalyst bed. A co-current flow of amine and ultra pure (BOC, >99.99%) H_2_ was maintained at total *GHSV* = 1 × 10^4^ h^−1^ with an inlet amine molar flow (*F*) of 3.5–6.1 mmol h^−1^. The H_2_ flow rate was monitored using a Humonics (Model 520) digital flowmeter. The molar Pd (*n*) to *F* ratio spanned the range 0.3 × 10^−3^ to 2.5 × 10^−3^ h. In blank tests, passage of MBA or DBA in a stream of H_2_ through the empty reactor did not result in any detectable conversion. The reactor effluent was frozen in a liquid N_2_ trap for subsequent analysis by capillary GC (Perkin-Elmer Auto System XL chromatograph equipped with a programmed split/splitless injector and FID), employing a DB-1 capillary column (i.d. = 0.33 mm, length = 50 m, film thickness = 0.20 μm). The effluent gas from the DBA reaction was bubbled through a water trap to absorb NH_3_ at ambient temperature [33]; pH was monitored (pH meter, Hanna Instruments) with time on-stream [34]. Reactant/product molar fractions (*x*_i_) were obtained using detailed calibration plots (not shown). Fractional conversion (*X*) is given by

2 $$X(-)=\frac{{{\left[ \text{reactant} \right]}_{\text{in}}}-{{\left[ \text{reactant} \right]}_{\text{out}}}}{{{\left[ \text{reactant} \right]}_{\text{in}}}}$$

with product selectivity (*S*_i_)

3 $${{S}_{\text{i}}}(\%)=\frac{{N}_{\text{i}}\cdot {x}_{\text{i}}}{\sum{{N}_{\text{i}}\cdot {x}_{\text{i}}}}\times 100$$

where [reactant]_in_ and [reactant]_out_ represent the concentration of amine entering (in) and leaving (out) the reactor and *N*_i_ is the stoichiometric coefficient for each product. Reactant consumption rate (*R*) was obtained from

4 $$R\,({\text{mol}_{\text{reactant}}}\,{{\text{h}}^{-1}}\,\text{mol}_{\text{Pd}}^{-1})\ =\frac{X\times F}{n}$$

Repeated reactions with different samples from the same batch of catalyst delivered raw data reproducibility and mass balances to within ±6%.

## Results and Discussion

### Production of DBA from MBA

**Fig. 2:**
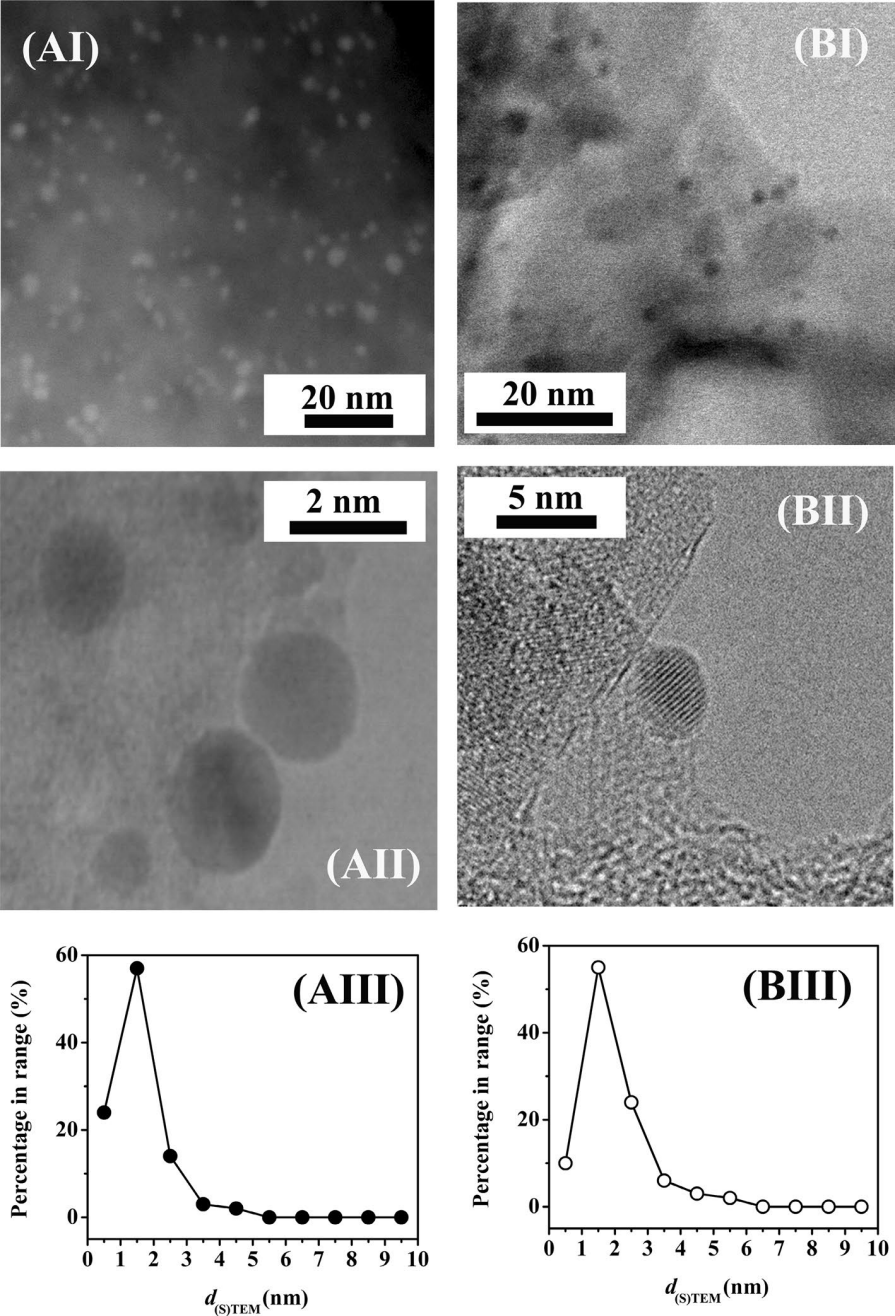
Representative (**I**) medium and (**II**) high magnification TEM/STEM images with (**III**) associated Pd size distribution for (**A**) Pd/C and (**B**) Pd/Al_2_O_3_

The existing literature suggests that formation of secondary amines from the corresponding mono-amine is a multi-step process (see Fig. 1), involving dehydrogenation (MBA → BI, step 1), condensation with NH_3_ release (BI + MBA → BBA, step 2) and hydrogenation (BBA → DBA, step 3) [10]. Reaction over Pd/C and Pd/Al_2_O_3_ resulted in the sole formation of DBA from MBA. In contrast, Kamiguchi and co-workers [10], using Pd/C to promote the gas phase condensation of MBA over 573–773 K, obtained BBA as principal and DBA as secondary product (*S*_DBA_ < 13%). High temperatures favour desorption of BBA [35] and the exclusivity to DBA that we achieve can be tentatively linked to the lower reaction temperature that allows transformation of BBA without desorption. In terms of catalytic activity, Pd/C (208 mol_MBA_ h^−1^ mol_Pd_^−1^) delivered a greater MBA transformation rate than Pd/Al_2_O_3_ (154 mol_MBA_ h^−1^ mol_Pd_^−1^). There is evidence in the literature that catalytic activity in hydrogenation [36] and dehydrogenation [37], critical for DBA generation (Fig. 1), is influenced by variations in Pd dispersion. The STEM/TEM images provided in Fig. 2, II) for Pd/C (Fig. 2A) and Pd/Al_2_O_3_ (Fig. 2B) reveal quasi-spherical particles at the nano-scale with a narrow (1–6 nm) size distribution (Fig. 2, III) and an equivalent mean (2.5–3.0 nm, Table 1). The observed differences in reaction rate can not be explained by variations in Pd size. In prior work [38], we demonstrated that condensation reactions in the conversion of butyronitrile to amines is enhanced by support acidity as has been noted for activated carbon [23, 24] and alumina [25–27]. Ambient temperature NH_3_ chemisorption coupled with TPD was used to quantify surface acidity. Ammonia release from Pd/C by TPD matched that chemisorbed and exceeded the amount recorded for Pd/Al_2_O_3_ (Table 1). Increased surface acidity facilitates condensation (Fig. 1, step 2), which can contribute to the observed higher DBA production rate over Pd/C.

**Table 1:** Physico-chemical properties of Pd/C and Pd/Al_2_O_3_

Catalyst	Pd/C	Pd/Al_2_O_3_
*d*_(S)TEM_ (nm)	2.5	3.0
NH_3_ chemisorbed (10^−5^ mol g^−1^)	94	52
NH_3_ TPD (10^−5^ mol g^−1^)	92	51
H_2_ chemisorbed (10^−2^ mol mol_Pd_^−1^)	27	22
H_2_ TPD (10^−2^ mol mol_Pd_^−1^)	849	102
SSA (m^2^ g^−1^)	870	145
Pd 3*d*_5/2_ BE (eV)	335.9	334.9

**Fig. 3:**
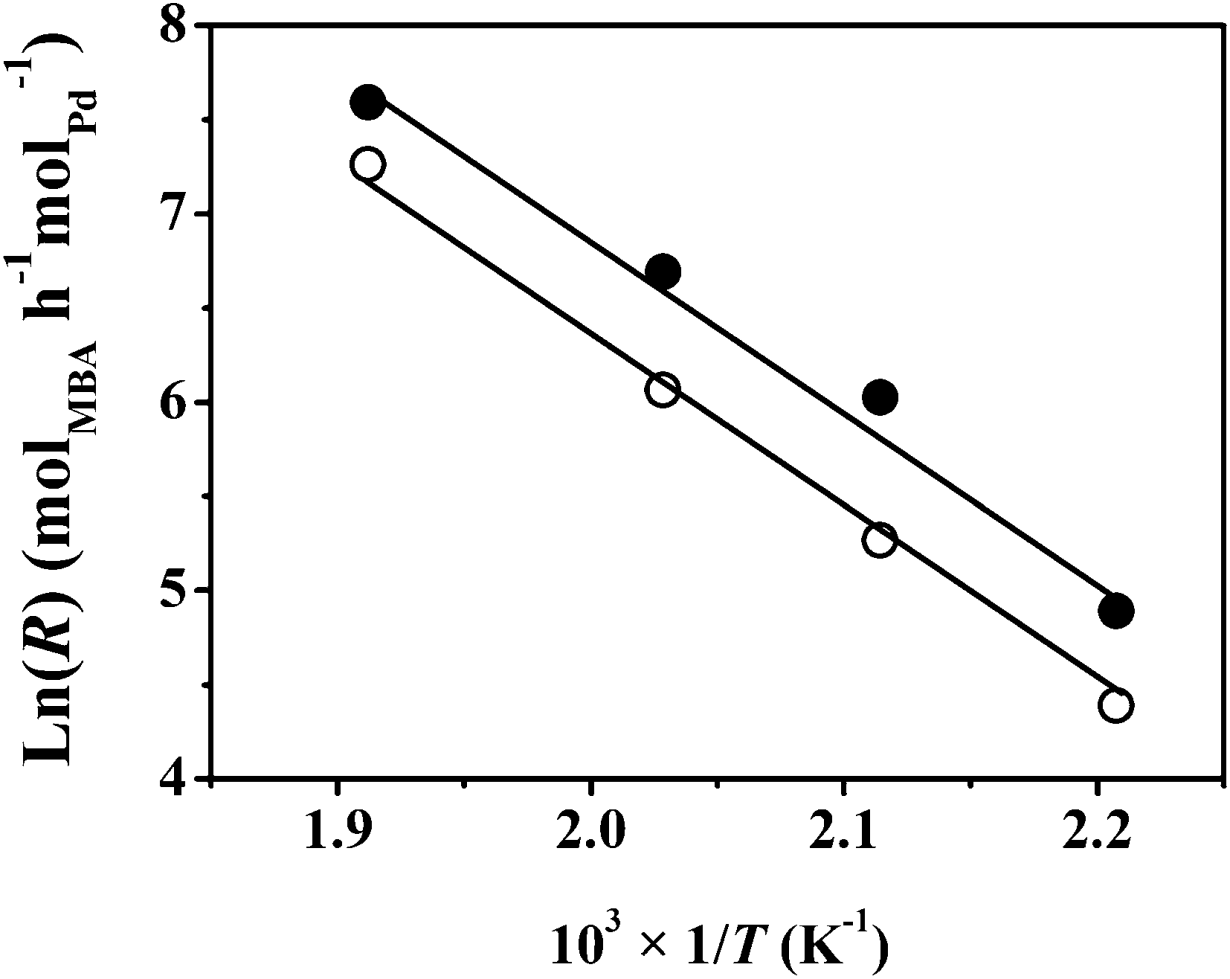
Arrhenius plots for the conversion of mono-butylamine (MBA) to di-butylamine (DBA) over Pd/C (*filled circle*) and Pd/Al_2_O_3_ (*open circle*)

As DBA formation involves hydrogenation of BBA (Fig. 1, step 3), availability of surface reactive hydrogen is an important parameter. Total surface hydrogen was evaluated by H_2_ TPD where in both cases H_2_ release far exceeded that measured in the chemisorption step (Table 1). This suggests hydrogen spillover, i.e. H_2_ dissociation at Pd sites with migration of atomic hydrogen to the support [39]. We can note studies that have established the occurrence of hydrogen spillover on activated carbon [40] and Al_2_O_3_ [41] supported Pd. Hydrogen desorption from Pd/C was appreciably greater than Pd/Al_2_O_3_ and can be linked to the higher SSA of Pd/C (Table 1), which can accommodate more spillover [40]. Reaction exclusivity was retained for the two catalysts over the temperature range 453 ≤ *T* ≤ 523 K and the associated Arrhenius plots are shown in Fig. 3. The resultant apparent activation energy (79 kJ mol^−1^) converged for both catalysts and is lower than that (100 kJ mol^−1^) reported for the conversion of mono-pentylamine to di-pentylamine [42]. The results establish that Pd/Al_2_O_3_ and Pd/C promote the gas phase continuous conversion of MBA solely to DBA. Pd/C delivered a higher DBA production rate, which can be associated with greater surface acidity (from NH_3_ chemisorption/TPD) that favours the condensation step and increased surface reactive hydrogen (from H_2_ TPD) that serves to promote BBA hydrogenation to DBA.

### Production of TBA from DBA

**Fig. 4:**
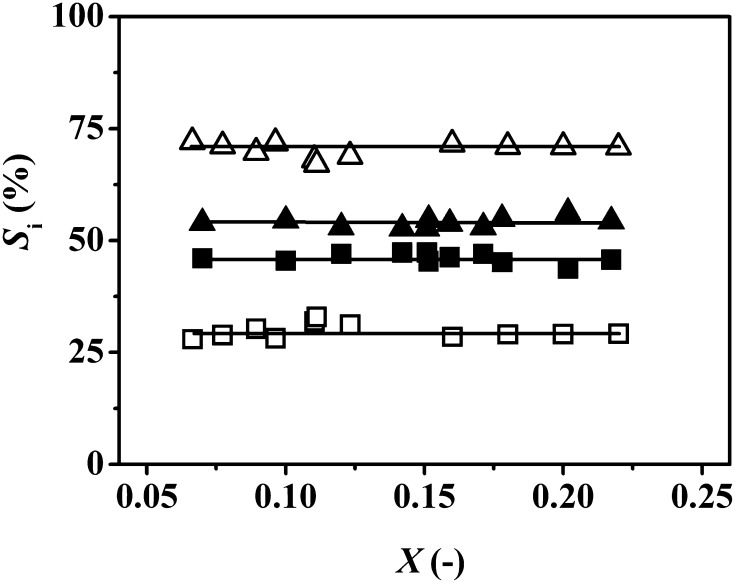
Selectivity (*S*_i_, %) to mono-butylamine [MBA (*filled square, open square*)] and tri-butylamine [TBA (*filled triangle, open triangle*)] as a function of di-butylamine (DBA) fractional conversion (*X*) for reaction over Pd/C (*solid symbols*) and Pd/Al_2_O_3_ (*open symbols*); *T* = 473 K; *P* = 1 atm

**Fig. 5:**
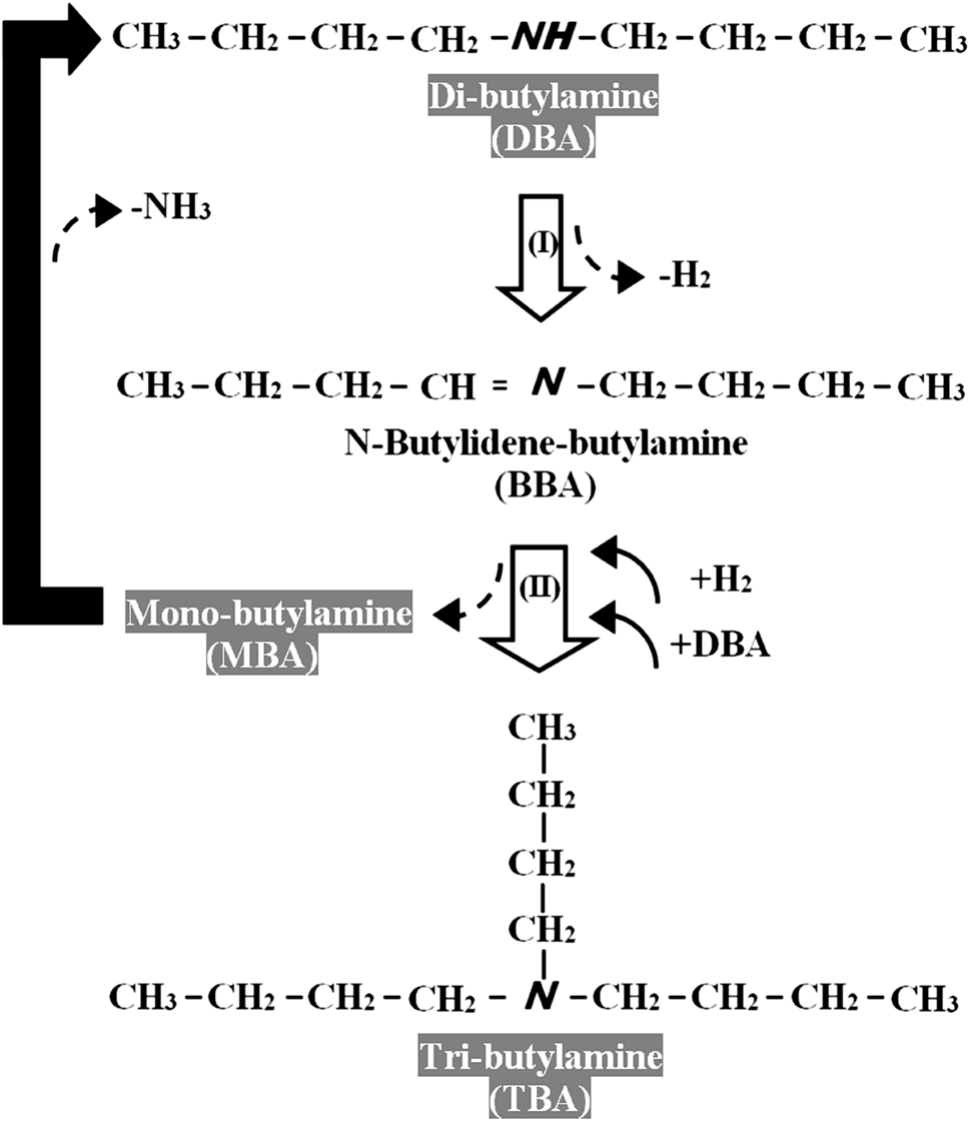
Proposed reaction scheme for the transformation of di-butylamine (DBA)

Given full selectivity to DBA from MBA, we explored the feasibility of continuous TBA production from DBA as feed. The conversion of DBA over both Pd catalysts generated a mixture of MBA and TBA, which is in line with a patent that serves as the only documented report of tertiary amine formation from secondary amines [9]. In terms of activity, Pd/C again delivered a significantly higher DBA consumption rate (Table 2). Selectivity was independent of DBA conversion (Fig. 4) where Pd/C generated equivalent amounts of TBA and MBA whereas Pd/Al_2_O_3_ promoted preferential formation of TBA. Based on the product distributions, we propose the reaction mechanism presented in Fig. 5, which involves dehydrogenation (DBA → BBA, step I) and hydrogen mediated disproportionation (BBA + DBA → MBA + TBA, step II). DBA adsorbs on Pd through the lone pair of electrons on N [43] resulting in bond polarisation (N^δ−^–C^δ+^), leading to dehydrogenation and the formation of BBA. Baiker [44] has established (by FTIR) formation of N-methylidene-methylamine from di-methylamine dehydrogenation (on Cu). Disproportionation of BBA with DBA generates TBA with the release of MBA. Xu et al. [45] working with CuO-NiO-PtO/*γ*-Al_2_O_3_ in gas phase operation under conditions similar to those used in this work (i.e. 473 K, 1 atm) demonstrated the formation of an aliphatic amine mixture (*N*-ethyl-*n*-butylamine, ethylamine, diethylamine, butylamine, dibutylamine and *N,N*-diethylbutylamine) via disproportionation of ethylamine + butylamine in hydrogen. Formation of TBA as product follows steps I/II in Fig. 5. The MBA that is generated can undergo combined dehydrogenation/condensation/hydrogenation as shown in Fig. 1 to form DBA. The catalytic results suggest that reaction over Pd/C predominantly follows steps I/II with equi-molar production of TBA and MBA. MBA formed on Pd/Al_2_O_3_ must undergo reaction (to generate DBA) with an overall greater relative enrichment of TBA in the product stream.

**Table 2:** Di-butylamine (DBA) consumption rate (*R*) and selectivity to tri-butylamine (*S*_TBA_) in single-, double- and triple- Pd/Al_2_O_3_ and Pd/C bed(s); *P* = 1 atm, *T* = 473 K

Catalyst	Pd/C		Pd/Al_2_O_3_	
	*R* (mol_DBA_ h^−1^ mol_Pd_^−1^)	*S*_TBA_ (%)	*R* (mol_DBA_ h^−1^ mol_Pd_^−1^)	*S*_TBA_ (%)
Single-bed	489	52	34	70
Double-bed	622	92	43	100
Triple-bed	698	100	–	–

**Fig. 6:**
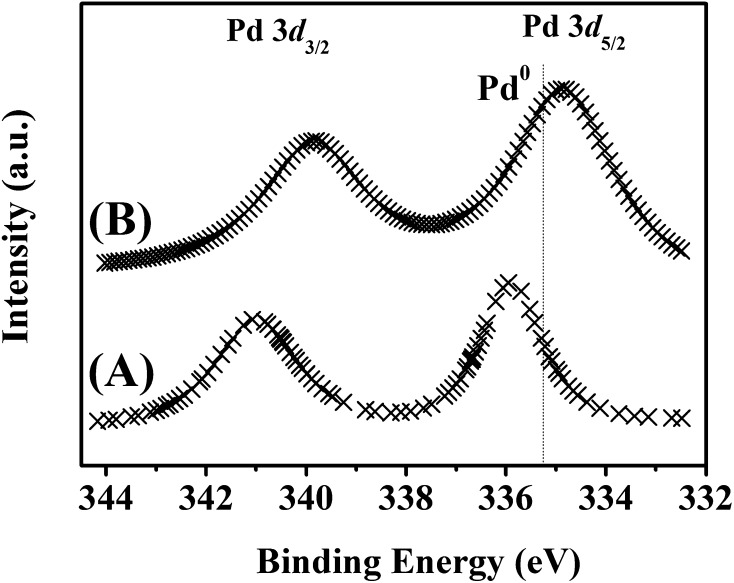
XPS spectra over the Pd 3*d* binding energy (BE) region recorded for ***A*** Pd/C and ***B*** Pd/Al_2_O_3_

Amine activation is governed by the electronic properties of the metal phase [46] that are, in turn, influenced by interactions with the carrier [32]. XPS analysis was conducted over the Pd 3*d* binding energy (BE) range in order to establish Pd charge and the resultant profiles are presented in Fig. 6; BE values are given in Table 1. Pd/C (Fig. 6A) exhibited a Pd 3*d*_5/2_ signal (at 335.9 eV) that is 0.7 eV higher than metallic Pd (335.2 eV) [47] indicating electron transfer to the carbon support with the generation of Pd^δ+^, as proposed elsewhere for nano-scale (4–12 nm) Pd on carbon [32, 48]. In contrast, Pd/Al_2_O_3_ (Fig. 6B) is characterised by a Pd 3*d*_5/2_ BE (334.9 eV) that is 0.3 eV lower than the metallic Pd reference, suggesting (partial) support → metal electron transfer. This is in accordance with the reported occurrence of electron-rich Pd^δ−^ (2–10 nm) on Al_2_O_3_ [32]. The nitrogen in DBA bearing two electron-donating *n*-butyl chains is more electron-rich than in MBA [49] with a consequent stronger interaction with Pd^δ+^ sites on the carbon support and competition for adsorption sites must result in a displacement of MBA from the surface by DBA. On the other hand, Pd^δ−^ sites on Al_2_O_3_ exhibit greater repulsion with respect to DBA relative to MBA where the latter is not displaced from the surface and can be transformed to DBA (Fig. 1). Monitoring the pH of an aqueous trap downstream of the reactor demonstrated greater alkalinity of the exhaust stream for reaction over Pd/Al_2_O_3_ (pH = 9.5) compared with Pd/C (pH = 7.8), which is consistent with NH_3_ production over the former via step 2 in Fig. 1.

In the proposed reaction scheme, surface reaction of DBA with BBA (step (II) in Fig. 5) results in TBA and MBA production. Operation of a second catalyst bed in series should facilitate conversion of MBA (to DBA) exiting the first bed leading to increased TBA yield. The experimental results obtained are provided in Table 2 where the same total mass of catalyst was divided into N (= 1–3) beds at the same inlet DBA and H_2_ flow rate. An increase in overall reaction rate and TBA selectivity was observed with increasing number of beds to attain target tertiary amine exclusivity in a two-bed arrangement for Pd/Al_2_O_3_ and triple Pd/C bed. This is the first time that full selectivity to a tertiary amine from a secondary amine feedstock has been reported. The higher TBA selectivity achieved over Pd/Al_2_O_3_ relative to Pd/C (Fig. 4) translated into a requisite lower number of catalyst beds in series to achieve full TBA selectivity.

## Conclusions

We have established exclusive formation of higher amines (DBA and TBA) from a lower amine (MBA and DBA, respectively) feedstock over nano-scale Pd (mean size = 2.5–3.0) supported on C and Al_2_O_3_ in gas phase continuous operation. Full selectivity in the conversion of MBA to DBA was attained over both catalysts with an associated apparent activation energy = 79 kJ mol^−1^. Reaction over Pd/C delivered a significantly higher DBA production rate, which is explained on the basis of a higher level of surface acidity (from NH_3_ chemisorption/TPD) and spillover hydrogen (from H_2_ TPD). Exclusive formation of TBA (from DBA) has been achieved over both catalysts where operation of beds in series resulted in higher TBA production rates. A reaction mechanism is proposed that accounts for our experimental observations. The results from this work can serve as a basis for an alternative clean and continuous production of higher amines from a lower amine feedstock.
